# Absence of Neuronal Response Modulation with Familiarity in Perirhinal Cortex

**DOI:** 10.1016/j.neuroscience.2018.10.020

**Published:** 2018-12-01

**Authors:** Liad J. Baruchin, Adam Ranson, Mark Good, Vincenzo Crunelli

**Affiliations:** a Neuroscience Division, School of Bioscience, Cardiff University, Cardiff, UK; b Neurosciences & Mental Health Research Institute, Cardiff University, Cardiff CF24 4HQ, UK; c Department of Psychology, Cardiff University, Cardiff, UK; d Department of Physiology and Biochemistry, Malta University, Msida, Malta

**Keywords:** ERPs, event-related potentials, MTL, medial temporal lobe, PRH, perirhinal cortex, VEP, Visual Evoked Potentials, memory, medial temporal lobe, ensemble recordings, visual evoked potentials

## Abstract

The perirhinal cortex (PRH) is considered a crucial cortical area for familiarity memory and electrophysiological studies have reported the presence of visual familiarity encoding neurons in PRH. However, recent evidence has questioned the existence of these neurons. Here, we used a visual task in which head-restrained mice were passively exposed to oriented gratings or natural images. Evoked potentials and single-unit recordings showed evoked responses to novelty in V1 under some conditions. However, the PRH showed no response modulation with respect to familiarity under a variety of different conditions or retention delays. These results indicate that the PRH does not contribute to familiarity/novelty encoding using passively exposed visual stimuli.

## Highlights

• LFP responses to images could be observed in the mouse PRH – which can be used to translate to human studies.
• Under passive head-restrained viewing condition no familiarity response modulation could be observed in the PRH.
• When many novel complex images are presented familiarity modulation could be observed as upstream as V1.

## Introduction

Many studies have provided evidence for a role for the medial temporal lobe (MTL) in familiarity memory, a form of recognition that signals whether a stimulus has been previously encountered (Squire et al., 2004, Eichenbaum et al., 2007, Ranganath et al., 2007). In particular, lesion studies in animals have indicated a major role for the perirhinal cortex (PRH), an area in the MTL, as necessary for object novelty memory (Ennaceur et al., 1996, Ennaceur and Aggleton, 1997, Winters et al., 2004). Moreover, studies in humans with lesions to the PRH have confirmed the importance of this region for recognition memory (Buffalo Reber and Squire, 1998). Indeed, experiments carried out mainly in monkeys, have identified a population of ‘familiarity-neurons’ within the PRH that respond to a visual stimulus by either decreasing or increasing their firing rate (Riches et al., 1991, Fahy et al., 1993, Zhu and Brown, 1995, Zhu et al., 1995).

In all studies investigating neural changes in PRH activity, the animals were familiarized to an object for extensive periods of time before neuronal recordings took place. For example, familiar objects were shown to rats every day for at least 5 days prior to the electrical recording (Zhu and Brown, 1995, Zhu et al., 1995). In most behavioral studies investigating the effects of PRH dysfunction on recognition memory, habituation to the sample object occurs over a relatively shorter period of time (c.f., Ennaceur et al., 1996). One aim of the current study was therefore to characterize changes in primary visual cortex V1 and the PRH cortex following relatively short periods of exposure to visually presented cues. While lesion studies have consistently highlighted a role for the PRH in object novelty/familiarity discriminations, other evidence has suggested this cortical region plays a more significant role in object processing when stimuli have overlapping features (Eacott et al., 2001; Bussey et al., 2003, Bussey et al., 2005, Cowell et al., 2006). A second aim of the current study, therefore, was to characterize V1 and PRH neural activity using simple gratings and more complex images of everyday objects. We used head-restrained animals in all conditions to minimize the impact of exploratory or motivational factors in influencing V1 or PRH responses to passively presented visual stimulation.

## Experimental procedures

### Animals

C57BL/6N mice, sourced from Charles Rivers were bred and maintained in-house on a C57/B6 background. The animals were kept on a normal 12:12-h light cycle, with lights on at 08:00, and were given access to food and water *ad libitum*. The housing room had a temperature of 19–21 °C and a relative humidity of 45–65%. Both female and male mice between the ages of 10 and 16 weeks were used for the experiments.

### Surgery

General anesthesia was induced in an induction box with a delivery of 4% isoflurane in 2 L/min 100% O2. The animal was then transferred to a stereotaxic frame where it received 3% isoflurane, which was gradually reduced to 2–1.5% during the course of the surgery, while ensuring that the animal remained anesthetized and maintained a stable breathing pattern. The depth of anesthesia was gauged during the surgery by checking the hind paw withdrawal and tail pinch reflexes. The temperature of the animal was monitored and maintained at 37 °C with a homeothermic heat blanket (#507220F, Harvard Apparatus, Kent, UK).

The animals head was shaved using electric clippers (Contura type HS61, Wella, UK). Then, the skin was disinfected with a povidone-iodine solution (Betadine, Betadine Inc., UK) to maintain a sterile surgical area. A paraffin-based eye lubricant (Lacrilube, Allegan Inc., USA) was applied to both eyes. Then, an incision was made to the scalp from the back of the skull to between the eyes using surgical scissors. The connective tissue covering the skull was carefully removed using sterile surgical swabs. Bregma and lambda were then identified as the intersection between the front horizontal and posterior horizontal sutures, respectively, and the vertical suture; and their stereotaxic coordinates were measured using a needle held by a stereotaxic manipulator arm. Then, the mice were implanted with electrodes in the areas of interest. For LFP acquisition, two depth electrodes were implanted, one in the visual cortex (+0.8 AP, 2.8 ML relative to lambda, −0.5 DV), and one in the perirhinal cortex (−3.3 AP, +4 ML relative to bregma −3.3 DV). A ground/reference screw was placed above the frontal sinus. For unit recordings, a silicone probe was mounted onto a mini-drive and was implanted in the PRH (∼−3.3 AP, ∼−4 relative to bregma, ∼−3.0 DV). Then, postoperatively the probe was slowly lowered into the recording area. The implantation sight was in a radius of about 100 µm around the intended implantation area, depending on brain vasculature. Two screws placed above the cerebellum were used as ground and reference.

After surgery, any loose skin flaps were sutured using braided 0.12-mm silk sutures. The wound area was then washed with saline an antiseptic powder (Battle Hayward and Bower Ltd, USA) was applied around the incision site. The anesthetic flow was then ceased and the animal left to breathe pure oxygen for a few seconds, until it regained its pinch reflex. Then, the animal was carefully removed from the stereotaxic frame and allowed to recover under heating light until it regained its righting reflex. It was moved back to the holding room. Animals were given a week to recover before any experimental procedure took place.

### Visual evoked potentials (VEP)

**Fig. 1:**
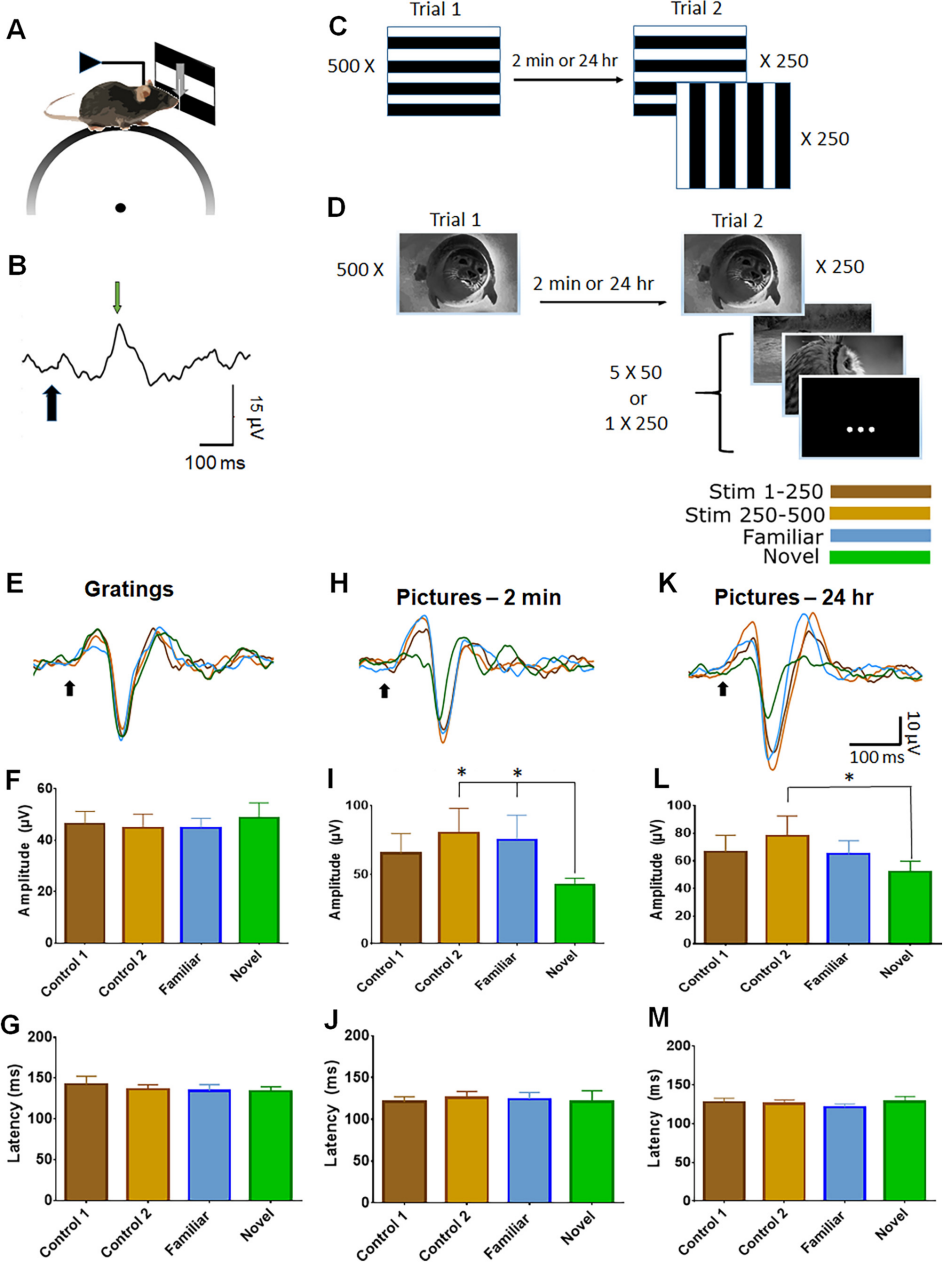
Experimental protocol and ERPs in primary visual cortex. (A) Experimental set-up. A mouse was fixed to a holder by an implanted head-plate, while being able to freely move on a running wheel. Visual stimuli were presented on a screen to the left of the mouse, while recording from the contralateral perirhinal cortex (PRH) or primary visual cortex (V1). (B) Typical ERP in the perirhinal cortex (PRH). The green arrow shows the first identifiable peak after the visual stimulus onset (black arrow). The ERP amplitude was calculated as the difference between this peak and the trough preceding it. The ERP latency was calculated as the time difference between the visual stimulus onset (black arrow) and the time to peak (green arrow). (C) The visual stimulation protocol consisted of 500 presentations of a grating of one orientation followed, after 2 min or 24 hr, by 250 presentations of a grating of the same orientation and 250 presentations of the same grating rotated by 90° (all gratings were presented at 100% contrast). (D) The natural image stimulation protocol consisted of 500 presentations of one natural image followed, after a retention interval of either 2 min or 24 hr, by 250 presentations of the same complex picture interleaved with either 50 presentations of 5 different novel complex pictures or by 250 presentations of one novel complex picture. (E) Average (*n* = 10) ERP in V1 in response to grating stimuli (black arrow marks stimulus onset). (F) Summary of mean ERP amplitude in V1 following grating stimuli. (G) Summary of mean ERP latency in V1 to grating stimuli. (H) Average (*n* = 13) ERP in V1 in response to object picture. (I) Summary of mean ERP amplitude in V1 following object picture. (J) Summary of mean ERP latency in V1 following object picture. (K) Average (*n* = 18) ERP in V1 in response to object picture stimuli with a 24-h retention interval. (L) Summary of mean ERP amplitude in V1 in response to object picture stimuli with a 24-hour retention interval. (M) Summary of mean ERP latency in V1 in response to object picture stimuli with a 24-hour retention interval. In F, G, I, J, L and M, Control 1 (brown) shows the mean (±SEM) response to the first 250 presentation within the first trial, and Control 2 (yellow) the average response for the last 250 presentations within the first trial; Familiar (blue) is the response for the 250 presentations of the familiar orientation in the second trial, and Novel (green) is the response for 250 presentations of a novel orientation in the second trial.

After implantation, rest and habituation, the animals were placed on linear treadmill, where they were head-restrained and free to run (Fig. 1A) as previously described (Ranson, 2017), while recording electrical activity from PRH and/or primary visual cortex (V1). The sessions were 20 minutes long and comprised of presentation of visual stimuli on the screen to the left of the mouse. The stimuli were presented for one second with one-second inter-stimulus interval. All the sessions were comprised of the presentation of 500 stimuli. The stimuli were horizontal and vertical gratings (Fig. 3.1A) or full-sized black and white pictures of different objects (Fig. 3.1B). The contrast and frequency of the gratings was chosen as the one eliciting the strongest response in previous studies (Frenkel et al., 2006, Cooke et al., 2015). Each trial consisted of 2 stages. At the first stage a stimulus, referred to as the ‘control’ stimulus – either a stationary grating or a picture – was presented 500 times. After a retention interval of either 2 min or 24 h, at the second stage the stimulus from the first stage, now designated the ‘familiar’ stimulus, was presented 250 times, interleaved with a novel stimulus (either a grating with a different orientation, or a novel picture). Under conditions in which pictures were used, another test consisted of a slightly different second stage, where the familiar stimulus was presented 250 times interleaved with 50 cases of different novel pictures. For the 2-minute retention period, the mouse stayed in the apparatus, with the screen turned on but without any stimulus. For the 24-hour retention interval, the mouse was returned to its home cage. During the inter-stimulus interval, the screen was a uniform and constant light gray color.

### Visual stimuli

Object images were drawn from a standardized image bank (Brodeur et al., 2012) Natural images were taken from a free stock photo website (http://www.freeimages.co.uk). Care was taken that images were not too similar when they were used for the same task, in terms of general contour and texture patterns. The images were resized to fit the entire presentation screen.

### VEP analysis

A custom-made automatic script was used to find the evoked potentials in both V1 and the PRH. All results were later verified visually. The average signal for all the trials in the different cases was averaged (250 trials) for each animal. For V1 (Fig. 3.2A), the most prominent trough was identified. The time of this trough relative to presentation onset was defined as the latency and the amplitude of the evoked potential was defined as the difference in amplitude between this trough and the peak directly preceding it. In the PRH (Fig. 3.2B), the first prominent peak was identified. The latency of this peak relative to stimulus onset was defined as the evoked-potential latency and its amplitude was defined as the difference between this peaks amplitude and the trough immediately preceding it.

### Movement analysis

Movement was recorded by a motion detector attached to the wheel on which the animal was placed. The movement recorded was the angular rotation of the wheel. To obtain an index of locomotor changes related to visual presentations, the movement that occurred within 1 s of stimulus presentation was divided by activity in the 1-s bin before the presentation for each stimulus.

## Results

Since work in humans has shown that event-related potentials (ERPs) are modulated by familiarity (Fell et al., 2002, Grunwald and Kurthen, 2006), we performed both ERPs and single-unit recordings (with a 32-site silicon probe; McCafferty et al., in press). Mice were familiarized with a stimulus by presenting it 500 times, with both a presentation time and the interval between successive stimuli of 1 s. After a retention interval of 2 min or 24 h, 250 presentations of either the familiar or a novel stimulus were interleaved. The visual stimuli were either simple gratings (Fig. 1C), or natural images objects (Fig. 1D). Neuronal responses were recorded during the two presentations. To determine whether the ERPs were modulated by familiarity, we compared the amplitude and latency of the ERPs (measured as described in Fig. 1B) of the first 250 presentations of a stimulus (Control 1) with the following 250 presentations of the same stimulus (Control 2) and the presentations of the familiar and novel stimuli after the retention interval. For multi-unit recordings, we compared the firing rate before and during stimulus presentation under the different conditions described above.

**Fig. 2:**
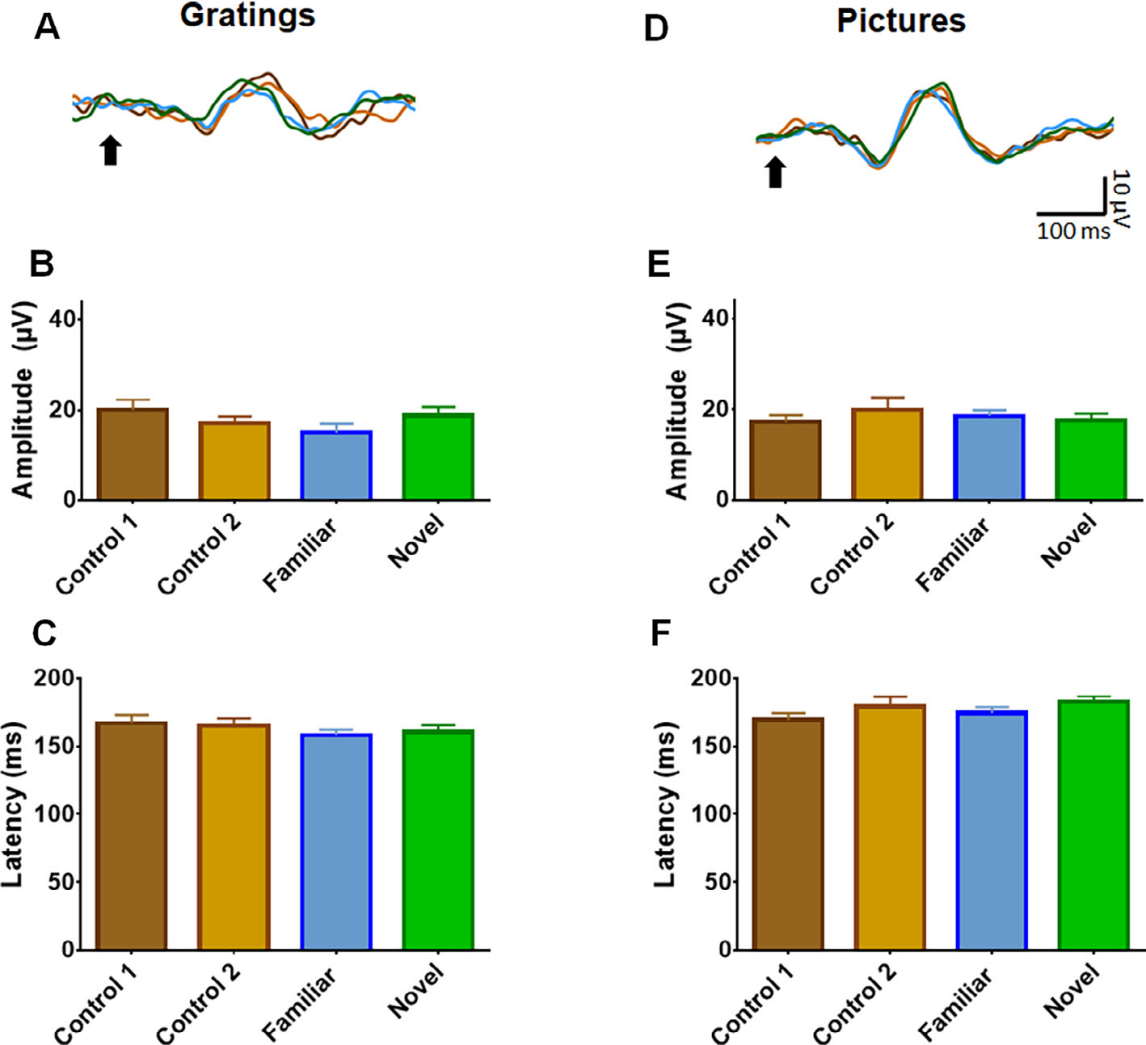
ERPs in the perirhinal cortex (PRH). (A) Average (*n* = 10) ERP in PRH in response to grating stimuli (B) Summary of mean ERP amplitude in PRH in response to gratings. (C) Summary of mean ERP latency in PRH in response to gratings. (D) Average (n-10) ERP in PRH in response to object picture stimuli. (E) Summary of mean ERP amplitude in PRH in response to natural images. (F) Summary of mean ERP latency in PRH in response to natural images. In B, C, E and F, Control 1 (brown) shows the mean (±SEM) ERP in response to the first 250 presentations within the first trial, and Control 2 (yellow) shows the average ERP in response for the last 250 presentations within the first trial. Familiar (blue) is the response for the 250 presentations of the familiar orientation in the second trial, and Novel (green) is the response for 250 presentations of a novel orientation in the second trial. Black arrow marks stimulus onset.

As expected ERPs were present in V1 (while simultaneously recording from PRH) indicating that both the gratings and the complex object pictures elicited neural activity in the early visual system (amplitude: −50.89 μV ± 5.66 μV, latency: 128.5 ms ± 5.22; ms; *n* = 10 mice; Fig. 1E, H, K). Both gratings and complex pictures evoked a robust ERP in the PRH (amplitude: 18.49 ± 0.95 μV; latency: 169 ± 5.74 ms; *n* = 10 mice) (Fig. 2A, D; these and subsequent quantitative data are mean ± SEM). We next tested for the emergence of familiarity/novelty-related differences in ERPs. We found no evidence for a difference in neural responses to familiar/novel stimuli, either in the amplitude of the grating ERP (*F*(3,27) = 2.11, *p* = 0.14, *n* = 10; ANOVA) (Fig. 2B), or in its latency (*F*(3,27) = 0.81, *p* = 0.49, *n* = 10) (Fig. 2C) in the PRH. Similarly, no change in these parameters was observed when animals were exposed to natural images (amplitude: *F*(3,33) = 0.66, *p* = 0.58, *n* = 12; latency: *F*(3,33) = 1.28, *p* = 0.29, *n* = 12) (Fig. 2E,F). In all cases, the mouse did not show any change in motor activity during the novel stimulus with either gratings (*F*(3,27) = 1.45, *p* = 0.25, *n* = 10), or pictures (*F*(3,33) = 1.26, *p* = 0.30, *n* = 12).

The absence of a reliable change in motor activity or neural activity in response to novelty might suggest the stimuli were not either processed effectively by the animal or the item designated as ‘novel’ became ‘familiar’ very rapidly during the procedure. We therefore increased stimulus ‘novelty’ during the test stage by randomly presenting 5 novel objects (each shown 50 times) on the second trial. Under these conditions, a familiarity effect was observed in V1, whereby the ERP elicited by novel stimuli was smaller in amplitude than those elicited by familiar stimuli and the Control 2 stimuli (*F*(3,36) = 5.28, *p* < 0.01, *n* = 13; Fig. 1H, I). In contrast, no change was detected in the latency (*F*(3,36) = 0.07, *p* = 0.81, *n* = 13; Fig. 1H, J). Despite stimulus novelty-related changes in V1, there was, nevertheless, no change in ERP amplitude (*F*(3,36) = 1.79, *p* = 0.17, *n* = 13) or latency (*F*(3,36) = 0.43, *p* = 0.70, *n* = 13) in the PRH (not shown). Again, there was no difference movement in response to the different stimulus categories (*F*(3,36) = 2.04, *p* = 0.125, *n* = 13).

Following damage to the PRH, rats show deficits in the NOE task only for intervals greater than approximately 15 min (Ennaceur et al., 1996, Ennaceur and Aggleton, 1997, Winters et al., 2004). This observation suggests that the PRH response to novelty/familiarity may be influenced by a long-retention interval. Therefore, to determine whether familiarity responses emerged with a retention interval, the same tests were repeated after a 24-h delay. Despite this longer interval, there was no change in the PRH ERP following familiar and novel (i) gratings (amplitude: (*F*(3,42) = 0.71, *p* = 0.93, *n* = 15 latency (*F*(3,42) = 0.32, *p* = 0.81, *n* = 15), (ii) complex pictures (amplitude: (*F*(3,27) = 1.23, *p* = 0.31, *n* = 10; latency *F*(3,27) = 0.08, *p* = 0.92, *n* = 10) or (iii) 5 novel complex pictures (amplitude: (*F*(3,42) = 1.81, *p* = 0.16, *n* = 15; latency: *F*(3,42) = 0.66, *p* = 0.53, *n* = 15). Interestingly, similarly to the short-delay experiments, in V1 the 5 novel natural images evoked a smaller ERP than the Control 2 stimuli (*F*(3,51) = 4.73, *p* < 0.01, *n* = 18; Fig. 1K, L), while no changes were observed in their latency (*F*(3,51) = 0.95, *p* = 0.37, *n* = 18; Fig. 1K, M). In all cases, there was no change in motor activity: (i) (*F*(3,42) = 1.95, *p* = 0.13, *n* = 15); (ii) (*F*(3,27) = 1.34, *p* = 0.27, *n* = 10). (iii) *F*(3,51) = 0.76, *p* = 0.52, *n* = 18).

**Fig. 3:**
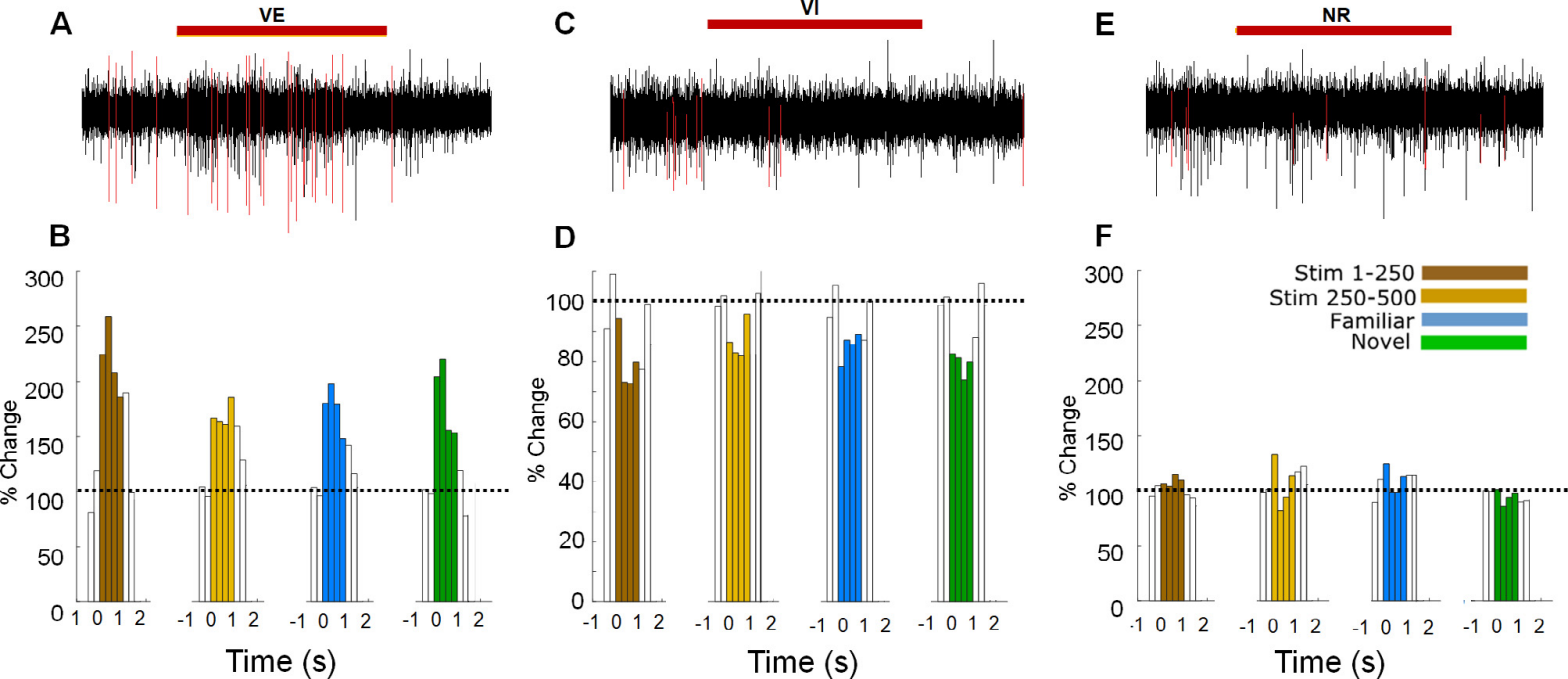
Activity of single neurons in perirhinal cortex (PRH) in responses to visual stimuli. (A, C, E) High-pass filter traces showing the typical response of three different PRH neurons before, during (marked by red horizontal bar) and after the visual stimulus (isolated spikes are marked in red). Examples of one PRH neuron that increased (left), one that decreased (center) and one that showed no change (right) in firing rate during visual stimulation (VE: visual excited, VI: visual inhibited, NR: visually non-responsive). (B) Peristimulus-time-histograms (PSTHs) from a typical PRH VE neuron that increased its firing-rate during stimulus presentation (ANOVA bin: *F*(4,3984) = 11.60, *p* < 0.001, interaction: *F*(9,3984) = 0.21, *p* = 0.89). (D) PSTHs form a typical PRH VI neuron in the PRH (ANOVA bin: *F*(4,3984) = 32.82, *p* < 0.001, interaction: *F*(9,3984) = 0.60, *p* = 0.79) that decreased its firing rate during stimulus presentation. (F) Example of an NR neuron (ANOVA bin: *F*(4,3984) = 1.02, *p* = 0.31, interaction: *F*(9,3984) = 0.84, *p* = 0.47) that did not change its firing rate during stimulus presentation. In the PSTHs, each bin is the mean frequency over a 250-ms time-window averaged over 250 presentations, and the dashed line marks the 100% baseline in the 500 ms preceding stimulus onset. Time 0 marks the start of stimulus onset.

Since previous studies have reported the presence of a subpopulation of ‘familiarity’ neurons in the PRH, it could be that the lack of changes in ERP observed in the present study resulted from the inability of our stimuli to engage a large enough neuronal ensemble to affect the ERP, or that subpopulations may have their activity modulated in opposing directions. Consequently, we next recorded simultaneously from many individual PRH neurons using a silicon probe, while the mouse was presented with various visual stimuli. Overall, 218 units in the PRH were isolated using klusta-kwik (Rossant et al., 2016) from gratings, pictures and 5 novel pictures’ (6 trials) conditions. On average, 19.2 ± 2.7% of the recorded neurons showed stimulus-related modulation of their firing in the PRH (Fig. 3A-D). The remaining neurons showed no change in their firing-rate in response to any stimulus (non-responsive-neurons; NR) (Fig. 3E,F). Averaged across all sessions, 68 ± 15% of responsive PRH neurons increased their firing rate during stimulus presentation (visually excited neurons: VE) (Fig. 3A,B), while the others decreased their firing-rate (visually inhibited neurons: VI) (Fig. 3B,C). There was no difference in the firing rate prior to stimulus presentation among NR, VE and VI neuronal populations (NR: 2.5 ± 0.5 Hz; VE: 2.6 ± 0.5 Hz; VI: 3.5 ± 1.1 Hz; *F*(2,204) = 1.142, *p* = 0.32; NR: 170, VE: 26, VI: 11). Interestingly, the response latency of the VE neurons was shorter than the VI neurons (*t*(55) = 3.375, *p* < 0.01). Importantly, none of the neurons showed familiarity-induced modulation in their response to stimuli.

## Discussion

The present study showed that both ERPs and single-neuron responses in the mouse PRH was not modulated by stimulus familiarity when passively exposed to simple gratings or more complex visual images.

One important difference between the current and previous studies that noted familiarity-related changes in PRH is the amount of exposure to the familiar cues. In this study, and in most NOE studies, the animal is typically exposed to the familiar stimulus over a relatively brief period (typically one trial). In previous electrophysiological experiments where a familiarity-modulated response in the PRH was observed, the animal was exposed to the stimulus over days prior to testing (Zhu and Brown, 1995, Zhu et al., 1995). Thus, it might be that the familiarity response reported in previous studies reflected extended exposure to a familiar stimulus. However, previous work has found that repeated exposure to a stimulus modulates both the ERP and multi-unit activity in V1 (Cooke et al., 2015). Similarly, in our experiments we have shown, that ERPs in V1 but not the PRH were modulated by familiarity, under some conditions. Thus, although there was evidence of familiarity-related changes in V1 in the current study, there were no changes observed in the PRH.

Previous work using c-Fos as an indirect measure of neural activity has revealed increased expression of protein in the PRH when rodents were exposed to novel objects, but not when familiar objects were presented in novel locations (Aggleton and Brown, 2005, Mendez et al., 2015). This evidence clearly suggests that the PRH is involved in some aspect of novelty processing. However, our own study suggests that this is not the case with passively exposed visual cues. Object-based recognition memory procedures differ from the current study in several ways. Perhaps one of the most important is the fact that object novelty paradigms involve an active process in which the animal samples the cue not only with the visual senses but also through other senses, such as olfactory and tactile information. It remains possible that the PRH is involved in familiarity/novelty discriminations but predominantly in situations involving an integrated multi-sensory representation of cues. On the other hand, other evidence has shown that lesions of the PRH caused disruption of recognition memory only whenever visual cues were available but not when olfactory or tactile information was available (Albasser et al., 2011b). This evidence suggests that the PRH is primarily involved in novelty/familiarity discriminations based on visual information. The absence of modulation of PRH activity (despite changes inV1 activity) when using passively presented visual cues is thus surprising; although not without precedent (Burke et al., 2012, Deshmukh et al., 2012).

One other important difference between the current method and object recognition paradigms is the opportunity in the latter to explore/sample different visual properties of an object. Although speculative, perhaps exploration of an object provides an opportunity to integrate visual information (features) about an object from different perspectives, thereby minimizing interference between objects (Gilbert and Kesner, 2003). The PRH may contribute to this higher level integrative process and the patterns of stimulation used in the present experiment may not have been sufficiently complex to engage this putative process. Although it is worth noting that we did vary stimulus complexity using gratings and more complex images of real-world objects, this did not reveal evidence of familiarity/novelty responses in the PRH. Finally, one other way in which the current study differs from standard tests of object familiarity in rodents is in the discrimination between novel and familiar cues presented concurrently on a trial. The comparison between familiar and novel cues may be an important component of the PRH neural response (but see Burke et al., 2012). Indeed, evidence has shown that while rats with lesions of the PRH were unable to perform simultaneous object novelty/familiarity discriminations, the same animals were able to perform a similar, successive, object novelty task (Olarte-Sanchez et al., 2015). In the latter condition, familiar or novel objects were presented separately and successively on test trials, as in the present study. Further work is clearly required to investigate the conditions under which the PRH is engaged by familiarity v novelty comparisons at the neural level.

In conclusion, the results of the present study are important in showing that neural activity in PRH cortex was not modulated by the familiarity/novelty of visual cues – despite changes in activity in V1. These results confirm and extend other evidence that PRH activity does not reflect a simple familiarity/novelty code but may (by inference) reflect more complex processes contributing to the integration of visual information and/or assigning a familiarity/novelty signal to a cue in a simultaneous visual discrimination.

## References

[b0005] Aggleton J.P., Brown M.W. (2005). Contrasting hippocampal and perirhinalcortex function using immediate early gene imaging. Q J Exp Psychol: Sect B.

[b0015] Albasser M.M., Amin E., Iordanova M.D., Brown M.W., Pearce J.M., Aggleton J.P. (2011). Perirhinal cortex lesions uncover subsidiary systems in the rat for the detection of novel and familiar objects. Eur J Neurosci.

[b0020] Brodeur M.B., Kehayia E., Dion-Lessard G., Chauret M., Montreuil T., Dionne-Dostie E., Lepage M. (2012). The bank of standardized stimuli (BOSS): comparison between French and English norms. Behav Res Methods.

[b9000] Buffalo E.A., Reber P.J., Squire L.R. (1998). The human perirhinal cortex and recognition memory. Hippocampus.

[b0030] Burke S.N. (2012). Representation of three-dimensional objects by the rat perirhinal cortex. Hippocampus.

[b0040] Bussey T.J., Saksida L.M., Murray E.A. (2003). Impairments in visual discrimination after perirhinal cortex lesions: testing “declarative” vs. “perceptual-mnemonic” views of perirhinal cortex function. Eur J Neurosci.

[b0045] Bussey T., Saksida L., Murray E. (2005). The perceptual-mnemonic/feature conjunction model of perirhinal cortex function. Q J Exp Psychol: Sect B.

[b0050] Cooke S.F., Komorowski R.W., Kaplan E.S., Gavornik J.P., Bear M.F. (2015). Visual recognition memory, manifested as long-term habituation, requires synaptic plasticity in V1. Nat Neurosci.

[b0055] Cowell R.A., Bussey T.J., Saksida L.M. (2006). Why does brain damage impair memory? A connectionist model of object recognition memory in perirhinal cortex. J Neurosci.

[b0060] Deshmukh S.S., Johnson J.L., Knierim J.J. (2012). Perirhinal cortex represents nonspatial, but not spatial, information in rats foraging in the presence of objects: Comparison with lateral entorhinal cortex. Hippocampus.

[b0065] Eichenbaum H., Yonelinas A.P., Ranganath C. (2007). The medial temporal lobe and recognition memory. Annu Rev Neurosci.

[b0070] Ennaceur A., Aggleton J.P. (1997). The effects of neurotoxic lesions of the perirhinal cortex combined to fornix transection on object recognition memory in the rat. Behav Brain Res.

[b0075] Ennaceur A., Neave N., Aggleton J.P. (1996). Neurotoxic lesions of the perirhinal cortex do not mimic the behavioural effects of fornix transection in the rat. Behav Brain Res.

[b0080] Fahy F.L.L., Riches I.P.P., Brown M.W.W. (1993). Neuronal activity related to visual recognition memory: long-term memory and the encoding of recency and familiarity information in the primate anterior and medial inferior temporal and rhinal cortex. Exp Brain Res.

[b0085] Fell J. (2002). The interaction of rhinal cortex and hippocampus in human declarative memory formation. Rev Neurosci.

[b0090] Frenkel M.Y., Sawtell N.B., Diogo A.C.M., Yoon B., Neve R.L., Bear M.F. (2006). Instructive effect of visual experience in mouse visual cortex. Neuron.

[b0095] Grunwald T., Kurthen M. (2006). Novelty detection and encoding for declarative memory within the human hippocampus. Clin EEG Neurosci.

[b0100] McCafferty C., David F., Venzi M., Lőrincz M.L., Delicata F., Atherton Z., Recchia G., Orban G., Lambert R.C., Di Giovanni G., Leresche N., Crunelli V. (2018). Cortical drive and thalamic feed-forward inhibition control thalamic output synchrony during absence seizures. Nat Neurosci.

[b0105] Mendez, Marta (2015). c-Fos expression correlates with performance on novel object and novel place recognition tests. Brain Res Bull.

[b0110] Ranganath C., Yonelinas A.P., Eichenbaum H. (2007). The medial temporal lobe and recognition memory. Annu Rev Neurosci.

[b0115] Ranson A. (2017). Stability and plasticity of contextual modulation in the mouse visual cortex. Cell Reports.

[b0120] Riches I.P., Wilson F.A., Brown M.W. (1991). The effects of visual stimulation and memory on neurons of the hippocampal formation and the neighboring parahippocampal gyrus and inferior temporal cortex of the primate. J Neurosci.

[b0125] Rossant C. (2016). Spike sorting for large, dense electrode arrays. Nat Neurosci.

[b0130] Squire L.R., Stark C.E.L., Clark R.E. (2004). The medial temporal lobe. Annu Rev Neurosci.

[b0140] Winters B.D. (2004). Double dissociation between the effects of peri-postrhinal cortex and hippocampal lesions on tests of object recognition and spatial memory: heterogeneity of function within the temporal lobe. J Neurosci.

[b0145] Zhu X.O., Brown M.W. (1995). Changes in neuronal activity related to the repetition and relative familiarity of visual stimuli in rhinal and adjacent cortex of the anaesthetised rat. Brain Res.

[b0150] Zhu X.O., Brown M.W., Aggleton J.P. (1995). Neuronal signalling of information important to visual recognition memory in rat rhinal and neighbouring cortices. Eur J Neurosci.

